# Metabolic Disorders in Chronic Lung Diseases

**DOI:** 10.3389/fmed.2017.00246

**Published:** 2018-01-18

**Authors:** Ourania Papaioannou, Theodoros Karampitsakos, Ilianna Barbayianni, Serafeim Chrysikos, Nikos Xylourgidis, Vasilis Tzilas, Demosthenes Bouros, Vasilis Aidinis, Argyrios Tzouvelekis

**Affiliations:** ^1^First Academic Department of Pneumonology, Hospital for Diseases of the Chest “Sotiria”, Medical School, National and Kapodistrian University of Athens, Athens, Greece; ^2^5th Department of Respiratory Medicine, Hospital for Diseases of the Chest “Sotiria”, Athens, Greece; ^3^Department of Internal Medicine, Section of Pulmonary Critical Care and Sleep Medicine, Yale School of Medicine, New Haven, CT, United States; ^4^Division of Immunology, Biomedical Sciences Research Center Alexander Fleming, Athens, Greece

**Keywords:** chronic lung diseases, metabolic disorders, comorbidities, metabolomics, pathogenetic pathways

## Abstract

Chronic lung diseases represent complex diseases with gradually increasing incidence, characterized by significant medical and financial burden for both patients and relatives. Their increasing incidence and complexity render a comprehensive, multidisciplinary, and personalized approach critically important. This approach includes the assessment of comorbid conditions including metabolic dysfunctions. Several lines of evidence show that metabolic comorbidities, including diabetes mellitus, dyslipidemia, osteoporosis, vitamin D deficiency, and thyroid dysfunction have a significant impact on symptoms, quality of life, management, economic burden, and disease mortality. Most recently, novel pathogenetic pathways and potential therapeutic targets have been identified through large-scale studies of metabolites, called metabolomics. This review article aims to summarize the current state of knowledge on the prevalence of metabolic comorbidities in chronic lung diseases, highlight their impact on disease clinical course, delineate mechanistic links, and report future perspectives on the role of metabolites as disease modifiers and therapeutic targets.

## Introduction

**Figure 1:**
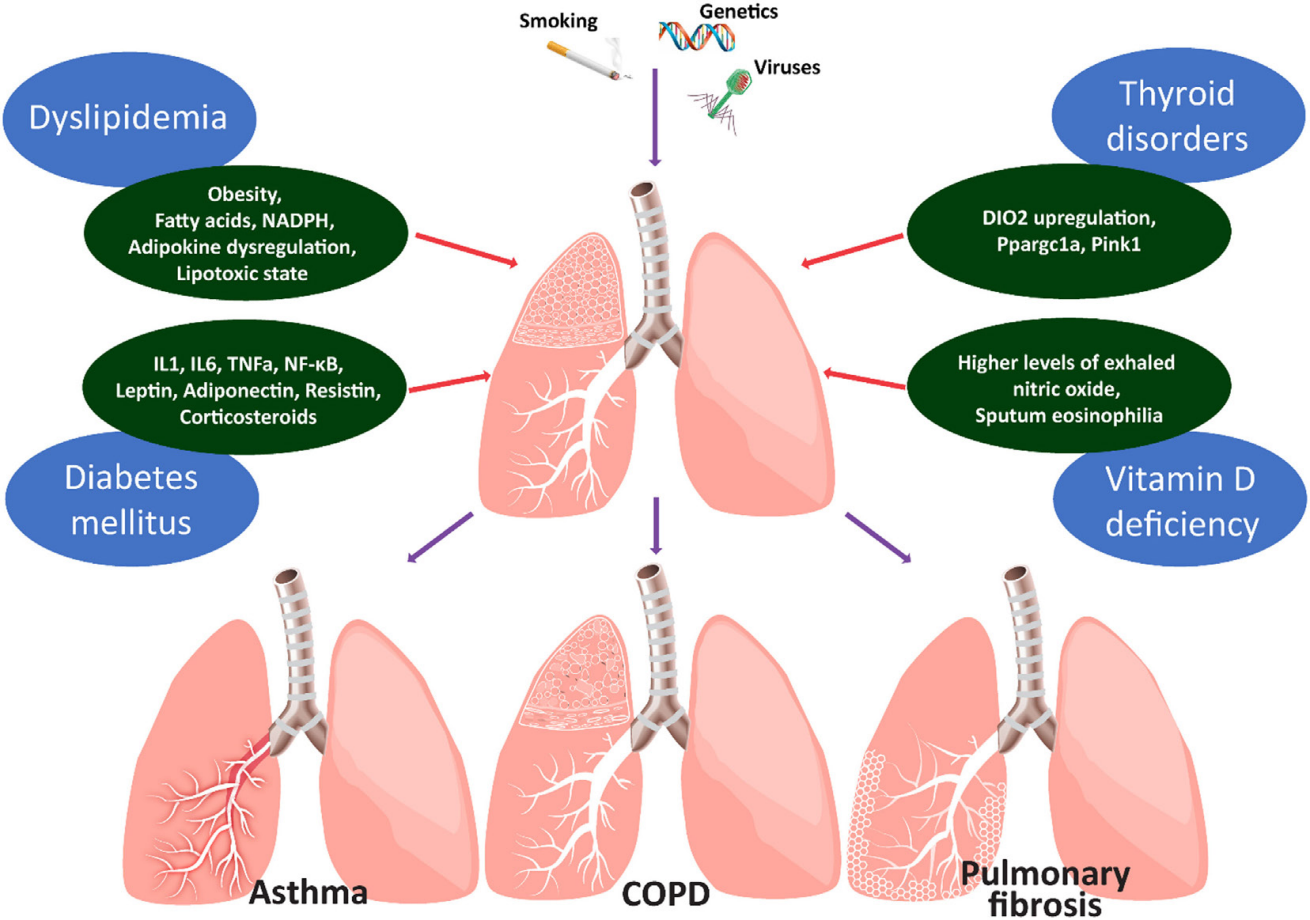
Figure depicts the main metabolic comorbidities of chronic lung diseases. Chronic lung diseases represent paradigms of the interplay between injurious environmental stimuli and genetic predisposition. Spillover of reactive oxygen species and pro-inflammatory mediators (IL1, 6, TNFa, NF-κB, leptin, adiponectin, and resistin) into the circulation may lead to insulin resistance in patients with chronic obstructive lung diseases. Chronic exogenous administration of corticosteroids may further affect the glycemic status through decreased insulin production and increased insulin resistance. Abdominal obesity of children with asthma and circulating fatty acids, adipokine dysregulation, and the lipotoxic state could represent a potential causal-effect relationship between asthma and dyslipidemia. Thyroid metabolism appears to affect alveolar epithelial cell homeostasis in the context of lung fibrosis. Hypothyroidism is associated with worst clinical outcomes in patients with IPF and IPF lungs are hypothyroid and display increased levels of type 2 iodothyronine deiodinase (DIO2). Aerosolized thyroid hormone administration blunts experimental lung fibrosis in two murine models through a mechanism that involves improvement of mitochondrial function and requires intact peroxisome proliferator-activated receptor gamma coactivator 1-alpha (PPARGC1A) and PTEN-induced putative kinase (PINK1) signaling pathways. The role of vitamin D in chronic lung inflammation is currently unknown. It is unclear whether this represents an epiphenomenon linked to other comorbidities or an underlying cause contributing to lung fibrogenesis. Clinical evidence suggests that patients with asthma and higher levels of exhaled nitric oxide or sputum eosinophilia were more likely to present with vitamin D deficiency.

Chronic lung diseases, including chronic obstructive pulmonary disease (COPD), asthma, and interstitial lung diseases (ILDs) constitute complex diseases with gradually increasing incidence, mortality, and major medical and financial burden (1). To this end, their management requires a comprehensive multidisciplinary and personalized approach, involving assessment of comorbid conditions (2). Most recently, evidence supports the role of endocrine system dysfunction in the pathogenesis of chronic lung diseases, and thus clinicians have integrated metabolic disorders in the Venn diagram of comorbidities of chronic lung diseases (3) (Figure 1). In particular, metabolic comorbidities exert a major impact on patients’ quality of life and mortality (1, 4). Diabetes mellitus, dyslipidemia, osteoporosis, and thyroid diseases (hypothyroidism and hyperthyroidism) are among the most commonly reported metabolic comorbidities in patients with chronic lung disease (5–8). Genes, age, and nutrition represent the three pillars of cellular metabolism and have been the topic of increasing scientific research in respiratory diseases (9, 10). This review article intends to summarize the most frequent metabolic comorbidities in association with their impact on chronic lung diseases, as well as to report future perspectives for their role in disease management.

## Metabolic Disorders and COPD

There is a compelling interest that COPD is a lung disease not only restricted to airway inflammation and remodeling (Table 1). Extrapulmonary comorbidities including metabolic disorders have been well recognized; yet, not fully understood (11). Current pathogenetic theories assume an interplay between systemic diffusion of local inflammation and consequences of age-related comorbid conditions which impact the lungs (11).

**Table 1:** Studies reporting prevalence of metabolic comorbidities in patients with chronic obstructive pulmonary disease (COPD).

Comorbidity	Prevalence	Prevalence in general population	Reference
Diabetes mellitus	10–18.7%	7–11.4%	Dursunoglu et al. (1), Framingham Heart Study Walter et al. (12), Laghi et al. (13)

Dyslipidemia	48.3%	18–46%	CONSISTE (14)

Osteoporosis	35.1% (9–69%)	5%	Graat-Verboom et al. (15)

Thyroid diseases	21.2% (hypothyroidism)	7.1% (hypothyroidism)	Terzano et al. (16, 17)
	32.2% (hyperthyroidism)	1.3–5% (hyperthyroidism)	

Osteoporosis represents the most frequent metabolic comorbidity of COPD (18). Bone disease occurs in 35.1% (range: 9–69%) of patients with COPD (15). Epidemiological studies showed a twofold increased risk of osteoporosis in patients with COPD compared with controls (19). Risk factors for developing osteoporosis in COPD are due to age, low body mass index, corticosteroid use, hypogonadism, or COPD-specific reasons (20). The latters include COPD functional stage, respiratory failure, severity of dyspnea, and COPD phenotype as determined by computed tomography scan (emphysematous versus non-emphysematous) (20). From a therapeutic point of view, it is unknown whether management of osteoporosis may exert a beneficial effect on disease outcome and thus prospective studies are sorely needed (21).

Metabolic syndrome is a complex disorder recognized clinically by the findings of abdominal obesity, elevated triglycerides, atherogenic dyslipidaemia, elevated blood pressure, and high blood glucose and/or insulin resistance. The metabolic syndrome is frequently encountered in patients with COPD in the context of coexisting systemic inflammation (6, 21–23). Prevalence of diabetes in COPD ranges between 10 and 18.7% (12, 13, 24). A study enrolling 103,614 women showed that patients with COPD had a 1.8 relative risk of diabetes development (25). An association between diabetes and impaired lung function has also been shown (13, 26–28). A plethora of pathogenetic commonalities between COPD and diabetes have been proposed; yet, data are still scarce (25). Spill over of reactive oxygen species and pro-inflammatory mediators (IL1, 6, TNFa, NF-κB, leptin, adiponectin, and resistin) into the circulation may lead to insulin resistance, chronic hyperglycemia, and increased lung collagen synthesis and deposition mediated by higher levels of advanced glycation products (29). Chronic exogenous administration of corticosteroids may further affect the glycemic status through decreased insulin production and increased insulin resistance due to increased muscle catabolism, lipolysis, and free fatty acids and thus enhance the vicious cycle of COPD, systemic inflammation, and dysglycemia (25, 30). Co-existing obesity may also contribute to insulin resistance as well as systemic inflammation through release of several pro-inflammatory mediators into the circulation, and thus perpetuate both local and systemic inflammation (29).

Abnormalities in lipoprotein metabolism have been associated with COPD (6); yet, relative mechanistic data are still scarce. A recent study reported dyslipidemia in 48.3% of patients with COPD and 31.7% of controls (14). The therapeutic criterion that statins are responsible for the reduction in death rate of patients with COPD by 36% highlights the interrelationship between COPD and dyslipidemia (31). Pleiotropic effects of statins such as anti-inflammatory and immunomodulatory properties should be further investigated in this setting (32).

The interest in the role of thyroid dysfunctions in patients with COPD has been recently revived (16, 17). Compelling evidence suggested that several characteristics of patients with COPD could potentially increase the risk of developing hypothyroidism or hyperthyroidism (33, 34). In particular, severity of airway obstruction, hypoxemia, and systemic inflammation might lead to subclinical hypothyroidism or overt hypothyroidism (35). Hypothyroidism has been associated with reduced respiratory muscle function, exercise capacity, and enhanced risk for sleep disordered breathing leading to inspiratory and expiratory weakness in patients with COPD (36). This weakness might be attributed to decreased expression of myosin heavy chains, phrenic nerve neuropathy, or decreased neuromuscular transmission (37). Hyperthyroidism has been also associated with impaired respiratory muscle function and exercise capacity in patients with COPD (36). Importantly, the reported substantial decrease of oxygen levels accompanied by carbon dioxide retention necessitates the evaluation of whether restoration of physiologic thyroid signaling may exert therapeutic effects on patients with COPD (16).

## Metabolic Disorders and Asthma

Asthma is among the most common chronic diseases worldwide (Table 2). The disease is poorly controlled despite available therapeutic regimens in a substantial minority of patients (38). Among factors impairing control of symptoms and treatment response are comorbid conditions including metabolic disorders (39, 40).

**Table 2:** Studies reporting prevalence of metabolic comorbidities in patients with asthma.

Comorbidity	Prevalence	Prevalence in general population	Reference
Dyslipidemia	18.38%	18% (age matched)	Heck et al. (39)

Diabetes mellitus	8.44%	7–11.4%	Heck et al. (39)

Abdominal obesity (severe asthma)	31% (children)	33.4–43.3%	Schatz et al. (41), Aranceta-Bartrina et al. (42)
	58% (adolescents-adults)		

Vitamin D deficiency	53.3% (children)	41.6%	Chinellato et al. (43), Devereux et al. (44), Forrest et al. (45)
	17% (adults)		

Recent evidence showed that the most common metabolic dysfunction was dyslipidemia, which occurred in 18.4% of asthmatic patients, while both types of diabetes mellitus—type 1 and 2—occurred in 8.44% of patients with asthma (39). A link between metabolic syndrome and functional indices of patients with asthma has been established both in children and young adults (46–49). Asthmatic children were more likely to have elevated triglycerides and acanthosis nigricans, a marker of insulin resistance leading to diabetes (49). Metabolic syndrome-induced lung impairment in asthma could be explained by the suppression in the complex effects of insulin and insulin receptors on the lung and the airways (50); however, the exact mechanism by which these receptors could affect the developing lung remains elusive (46). Circulating levels of fatty acids and the lipotoxic state inducing innate immune responses *via* multiple inflammatory mechanisms, such as pattern recognition receptor activation or intracellular signaling pathways might represent a link between asthma and dyslipidemia; yet, data are still scarce (47, 51). Abdominal obesity, another key feature of metabolic syndrome, has been associated with lung function impairment in asthma (47). The incidence of asthma almost doubled in obese subjects, while obesity represented a risk factor for severe asthma (41, 52). Experimental data showed that obese patients with asthma had a higher expression of inflammatory markers and adipokines in visceral fat, and thus adipokine dysregulation was suggested as a possible a mechanism leading to obesity-mediated airway changes in asthma (53, 54).

Osteoporosis has been linked with asthma as well, mainly due to chronic corticosteroid therapy (55–57); yet musculoskeletal complications of inhaled corticosteroids are highly debatable (55, 58). Lower bone mineral density in adult asthmatic patients using inhaled glucocorticoids compared to untreated controls has been described (57, 59, 60), even though this finding was not uniform (61, 62). A dose response relationship between use of oral glucocorticoids with risk of fracture in patients and asthma has been extensively validated (55, 63).

An epidemiologic and mechanistic interplay between vitamin D deficiency and asthma exacerbations has been reported (38, 64). Decreased levels of serum 25-hydroxyvitamin D were associated with increased prevalence and rates of hospitalization along with reduced lung function and increased airway hyperresponsiveness in asthmatic children (44, 65). Studies found that the prevalence of vitamin D deficiency was 53.3% among asthmatic children and 17% among asthmatic adults, respectively (43, 44). Other studies showed no differences in the mean vitamin D levels between asthmatics and healthy controls, while vitamin-D deficiency was strongly associated with sputum eosinophilia, higher levels of exhaled nitric-oxide, and lung function impairment. These evidence indicate that low vitamin D levels may potentially contribute to asthma exacerbation in those patients already susceptible to disease development (66). Vitamin D deficiency has been mechanistically linked with exaggerated airway smooth muscle contractility, particularly in cases of steroid-refractory asthma and asthma exacerbations (67). A potential anti-inflammatory role of vitamin D, a steroid hormone, has also been suggested through suppression of the Th2 immunologic response (65).

To this end, supplementation therapeutic strategies have been applied in large cohorts of asthmatic patients with encouraging results (68–70). A recent Cochrane meta-analysis of seven trials including 435 children, and two studies including 658 adults stated that oral vitamin D supplement reduced the risk of severe asthma exacerbations requiring hospitalization from 6% in the control group to 3% in the treatment arm (71). Despite promising therapeutic efficacy there is much to be learned, given that the aforementioned data mainly arise from just three trials and it is currently unknown whether this therapeutic effect can be expanded to all asthmatic patients or in those with low baseline levels of vitamin D.

## Metabolic Disorders and ILDs

Interstitial lung diseases constitute a group of diffuse parenchymal lung disorders, associated with substantial morbidity and mortality (72–76) (Table 3). Idiopathic pulmonary fibrosis (IPF) and sarcoidosis are among the most common ILDs (77, 78). The role of metabolic disorders in ILDs has been recently revived leading to studies investigating possible therapeutic targets for patients with ILDs (4, 79, 80).

**Table 3:** Studies reporting prevalence of metabolic comorbidities in patients with ILDs.

	Comorbidity	Prevalence	Prevalence in general population	Reference
IPF	Diabetes mellitus	10–39%	11.4%	British study (81), Japanese study (82), American study (83)
	Dyslipidemia	11–21.7%	18–46%	Enomoto et al. (82), Kaddah et al. (84), Sherbini et al. (85)
	Hypothyroidism	16.8% (13% men, 28% women)	7.1%	Oldham et al. (86)

Sarcoidosis	Thyroid diseases	13.1%	4%	Nowinski et al. (87)
	Diabetes mellitus	7.4%	7%	Nowinski et al. (87)
	Osteoporosis	5.7%	5%	Nowinski et al. (87)
	Hypercalcemia	10–15%	2%	Saidenberg-Kermanac’h et al. (88), Press et al. (89)

*ILDs, interstitial lung diseases; IPF, idiopathic pulmonary fibrosis*.

Diabetes mellitus represent the most frequently encountered endocrine comorbidity in patients with IPF (4, 79, 90–93). The prevalence of diabetes in patients with IPF ranged from 10 to 39% (81–83). A potential association between IPF and diabetes could be attributed to complications from chronic corticosteroid therapy (81, 82, 94). The impact of diabetes on disease mortality still remains elusive and controversial (82, 95, 96).

Thyroid disorders have been recently implicated as common comorbid conditions in patients with IPF (4, 80, 93, 97–99). Two recent studies demonstrated higher prevalence of hypothyroidism among patients with IPF (16.8% of subjects with IPF and 7.1% of control subjects). Interestingly, 13% of men and 28% of women were affected (86, 93). An interesting observation was that presence of hypothyroidism was associated with worse outcomes in patients with IPF (86). Interestingly, our study group identified that type 2 iodothyronine deiodinase (DIO2), the enzyme that converts T4 to active T3, was upregulated in the lungs of patients with IPF and particularly in alveolar epithelial cells, the metabolically active cells of the lung (100). DIO2 induction potentially reflected a compensatory response in order to boost local conversion of T4 to T3 to enhance the metabolic state of alveolar epithelial cells under stress conditions, considering that DIO2 knockout mice exhibited enhanced fibrotic responses to bleomycin. Intriguingly, experimental data showed that aerosolized thyroid hormone administration exerted anti-fibrotic effects in two experimental models of lung fibrosis through a mechanism that involved improved mitochondrial function and mitophagy (100). Same results were observed with sobetirome, a thyroid-mimetic agent that acts through activation of thyroid hormone signaling by selective binding to thyroid hormone receptor (100). Further studies exploring the effect of thyroid hormone administration in patients with IPF are greatly anticipated.

With regard to dyslipidemia, the reported prevalence in patients with IPF ranges between 11 and 21.7% (84, 85). Interestingly, Enomoto et al. recorded dyslipidemia in 19.2% of patients with IPF and 46% in the control group (82). The exact role of dyslipidemia, elevated levels of fatty acids, and oxidative stress *via* nicotinamide adenine dinucleotide phosphate oxidase activation in the pathogenesis of pulmonary fibrosis remains to be addressed (101).

Sarcoidosis is a multisystem inflammatory disease characterized by the presence of non-caseating granulomas in the affected organs (102–104). Lungs are affected in more than 90% of patients with sarcoidosis (104, 105). Data on the impact of metabolic comorbidities in sarcoidosis is limited and have mainly focused on calcium metabolism (87). In particular, 13.1% of patients with sarcoidosis were also diagnosed with thyroid diseases, 7.4% with diabetes, and 5.7% with osteoporosis, respectively (87). Age and multiorgan involvement of sarcoidosis represented risk factors for metabolic comorbidities (106). Finally, a case-control study of 111 patients with sarcoidosis suggested a potential association between autoimmune thyroid disorders and sarcoidosis (107, 108).

Hypercalcemia and hypercalcuria occur in a small, but significant number of patients with sarcoidosis, but if present, they constitute indication for treatment (103, 109, 110). Although earlier studies reported that hypercalcemia was present in 2–63% of patients with sarcoidosis, recent data show that the true prevalence is between 10 and 15% (88). Increased activity of the 1-alpha hydroxylase enzyme of tissue macrophages has been suggested to have a crucial role for the elevation of levels of 1,25-dihydroxyvitamin D3 (calcitriol), the hormonally active metabolite of vitamin D, which is responsible for hypercalcemia (111, 112). Bell et al. were the first to demonstrate elevated serum calcitriol levels in patients with sarcoid hypercalcemia (112). Since then, our understanding for the ideal therapeutic management of hypercalcemia associated with sarcoidosis has been significantly increased (113). Glucocorticoids have been used as first-line therapy in the management of hypercalcemia associated with sarcoidosis; yet with major complications that should be treated cautiously. Steroid-sparing agents including azathioprine and methotrexate have widely used in advanced disease stages or steroid-refractory cases with controversial results (113, 114). Finally, the investigation of the role of biphosphonates and especially zoledronic acid in sarcoid hypercalcemia is currently under investigation (113, 115).

## Metabolomics in Chronic Lung Diseases

For the past few years, the field of cellular bioenergetics and metabolism and their implication in the pathogenesis of chronic lung diseases has received much of attention. The term “metabolomics” refers to the systematic investigation of metabolic pathways (metabolome) and biochemical compounds created in a living system, called metabolites at a specific timepoint (116). Currently, quantification of metabolites of a biological system is performed by two techniques: mass spectrometry and nuclear magnetic resonance spectroscopy (NMR) (116). Urine, plasma, and lung tissue represent excellent biological specimens to study metabolomics with urine being the most promising one, because of its ease of collection, low cell, and protein content and rich chemical composition (117). Exhaled breath condensate is an easily accessible biomarker tool to study metabolome of the airway lining fluid, yet it presents with major limitations, since it is affected by several confounding factors, such as age, sex, smoking, temperature, humidity, and oral cavity contamination (117). Preliminary studies have shown that urine metabolomics profile could be used as reliable biomarker to diagnose heterogeneous syndromes with complex underlying pathogenesis, such as asthma (118) and COPD (117), and most importantly to differentiate asthma from COPD based on their metabolomic profile (119). Exhaled breath condensate leukotrienes have been used to distinguish asthmatic patients from controls (120). COPD patients exhibit abnormal muscular bioenergetics (121) and impaired microbiome-related metabolites (122) as indicated by increased plasma levels of branched-chain amino acids and urinary levels of hippurate and formate, respectively. Metabolomics profile has been also used to distinguish COPD patients with different phenotypes based on severity of functional impairment and the presence of emphysema and cachexia (123). Disrupted glycolysis, enhanced fatty acid accumulation, increased lactic acid, and lactate dehydrogenase production, as well as haem degradation have been identified as major events of impaired mitochondrial metabolism in both IPF and COPD patients (124–127). In addition, IPF lungs showed disrupted glutathione synthesizing pathway and consequently increased oxidant burden. Increased formation of proline, a key substrate for collagen biosynthesis, from ornithine through activation of ornithine aminotransferase has been also shown in IPF lungs (126). Interestingly, increased levels of ornithine aminotransferase have been negatively correlated with functional indices of disease severity including FVC (126). Intermediate metabolites of glycolysis including lactic acid have been shown to activate the TGF-β pathway inducing myofibroblast differentiation (127). Glycolytic reprogramming, a form of Warburg effect seen in cancer cells, has been recently implicated in fibroblasts to myofibroblasts differentiation. Inhibition of glycolysis exerted therapeutic effects in experimental lung fibrosis, highlighting a novel therapeutic area by shifting the metabolic requirements of key cellular components toward oxidative phosphorylation (128). The role of impaired mitochondrial metabolism in the pathogenesis of lung fibrosis has been recently demonstrated by a study showing that IPF lungs exhibit alveolar epithelial cells with damaged and dysfunctional mitochondria due to downregulated levels of PINK1, the master transcription factor of mitophagy (129). The cardinal role of mitochondrial metabolism in alveolar epithelial cell apoptosis in the context of lung fibrosis has been also highlighted by a recent publication from our study group showing therapeutic effects of aerosolized hormone administration in experimental lung fibrosis through enhancement of mitochondrial bioenergetics, as reported above (100). Interestingly, the concept of impaired mitophagy-mediated lung fibrosis has been recently suggested for lung macrophages and fibroblasts; yet, on a cell-specific manner (130–132). The above findings have also proven given human relevance, that products of fibroblasts’ mitochondrial metabolism including mitochondrial DNA (mtDNA) have been recently shown to serve as prognosticators of IPF mortality (133). The above preclinical studies highlight the importance of therapeutic restoration of the disrupted metabolome and the use of circulating metabolites as biomarkers of disease prognosis and treatment response.

## Future Perspectives and Concluding Remarks

There is increasing evidence that ameliorating the metabolic profile of a subgroup of patients with chronic lung diseases could have an impact on disease clinical course. To this end, extensive monitoring of metabolic alterations involving glucose metabolism, lipids and thyroid hormones signaling, calcium, and vitamin-D should be implemented in the everyday clinical practice of patients with chronic lung diseases. The exact role of vitamin D in patients with chronic lung diseases and its role in disease management remain to be addressed. In view of the current disappointing status of the therapeutic compounds targeting the extracellular matrix (90), novel drug discoveries aim to protect the epithelium or disrupt myofibroblast differentiation through restoration of physiologic cellular metabolism. Studies on the role of aerosolized thyroid hormone administration in patients with IPF are greatly anticipated. In the, not so distant, future metabolomics could represent the missing line that will connect the dots of translational research to the clinical setting. The idea of investigating the metabolic profile to stratify patients based on disease prognosis and treatment response has been applied in diabetes mellitus or hyperlipidemia for decades. In parallel with smoking and physical activity, diet is an important contributor for prevention of disease development and progression. Ultimately, the clinical judgment will still prevail, but molecular tools can complement clinical criteria. Similarly, to what is happening in diabetes, dyslipidemia, liver, and renal diseases, specific nutritional regimens based on the patient’s metabolomic profile may exert a beneficial impact on disease progression and mortality in chronic lung diseases. Cellular metabolism is too precious to be underestimated.

## Author Contributions

OP wrote the manuscript along with TK. The manuscript was supervised and significantly modified by AT. All authors offered intellectual contribution and approved the manuscript.

## Conflict of Interest Statement

The authors declare that the research was conducted in the absence of any commercial or financial relationships that could be construed as a potential conflict of interest.

## References

[1] Dursunoglu N Kokturk N Baha A Bilge AK Borekci S Ciftci F Comorbidities and their impact on chronic obstructive pulmonary disease. Tuberk Toraks (2016) 64(4):289–98. 10.5578/tt.2245 28393718

[2] Spagnolo P Tzouvelekis A Maher TM. Personalized medicine in idiopathic pulmonary fibrosis: facts and promises. Curr Opin Pulm Med (2015) 21(5):470–8. 10.1097/MCP.0000000000000187 26132817

[3] Farag AM Head and neck manifestations of endocrine disorders. Atlas Oral Maxillofac Surg Clin North Am (2017) 25(2):197–207. 10.1016/j.cxom.2017.04.011 28778308

[4] Raghu G Amatto VC Behr J Stowasser S. Comorbidities in idiopathic pulmonary fibrosis patients: a systematic literature review. Eur Respir J (2015) 46(4):1113–30. 10.1183/13993003.02316-2014 26424523

[5] Soriano JB Visick GT Muellerova H Payvandi N Hansell AL. Patterns of comorbidities in newly diagnosed COPD and asthma in primary care. Chest (2005) 128(4):2099–107. 10.1378/chest.128.4.2099 16236861

[6] Naik D Joshi A Paul TV Thomas N. Chronic obstructive pulmonary disease and the metabolic syndrome: consequences of a dual threat. Indian J Endocrinol Metab (2014) 18(5):608–16. 10.4103/2230-8210.139212 25285275 PMC4171881

[7] Oppedal RJ Khan DA Brown ES Hypothyroidism in patients with asthma and major depressive disorder. Prim Care Companion J Clin Psychiatry (2007) 9(6):467–8. 10.4088/PCC.v09n0611d 18185831 PMC2139917

[8] Mannino DM Thorn D Swensen A Holguin F. Prevalence and outcomes of diabetes, hypertension and cardiovascular disease in COPD. Eur Respir J (2008) 32(4):962–9. 10.1183/09031936.00012408 18579551

[9] Schols AM. Nutritional advances in patients with respiratory diseases. Eur Respir Rev (2015) 24(135):17–22. 10.1183/09059180.00010914 25726550 PMC9487767

[10] Lopez-Otin C Blasco MA Partridge L Serrano M Kroemer G. The hallmarks of aging. Cell (2013) 153(6):1194–217. 10.1016/j.cell.2013.05.039 23746838 PMC3836174

[11] Mullerova H Agusti A Erqou S Mapel DW. Cardiovascular comorbidity in COPD: systematic literature review. Chest (2013) 144(4):1163–78. 10.1378/chest.12-2847 23722528

[12] Laghi F Adiguzel N Tobin MJ. Endocrinological derangements in COPD. Eur Respir J (2009) 34(4):975–96. 10.1183/09031936.00103708 19797671

[13] Walter RE Beiser A Givelber RJ O’Connor GT Gottlieb DJ. Association between glycemic state and lung function: the Framingham Heart Study. Am J Respir Crit Care Med (2003) 167(6):911–6. 10.1164/rccm.2203022 12623860

[14] de Lucas-Ramos P Izquierdo-Alonso JL Rodriguez-Gonzalez Moro JM Frances JF Lozano PV Bellon-Cano JM Chronic obstructive pulmonary disease as a cardiovascular risk factor. Results of a case-control study (CONSISTE study). Int J Chron Obstruct Pulmon Dis (2012) 7:679–86. 10.2147/COPD.S36222 23055717 PMC3468057

[15] Graat-Verboom L Wouters EF Smeenk FW van den Borne BE Lunde R Spruit MA. Current status of research on osteoporosis in COPD: a systematic review. Eur Respir J (2009) 34(1):209–18. 10.1183/09031936.50130408 19567604

[16] Terzano C Romani S Paone G Conti V Oriolo F. COPD and thyroid dysfunctions. Lung (2014) 192(1):103–9. 10.1007/s00408-013-9537-6 24281671

[17] Sarinc Ulasli S Bozbas SS Ozen ZE Ozyurek BA Ulubay G. Effect of thyroid function on COPD exacerbation frequency: a preliminary study. Multidiscip Respir Med (2013) 8(1):64. 10.1186/2049-6958-8-64 24079533 PMC3845712

[18] Fouda MA Alhamad EH Al-Hajjaj MS Shaik SA Alboukai AA Al-Kassimi FA. A study of chronic obstructive pulmonary disease-specific causes of osteoporosis with emphasis on the emphysema phenotype. Ann Thorac Med (2017) 12(2):101–6. 10.4103/atm.ATM_357_16 28469720 PMC5399683

[19] Chen SJ Liao WC Huang KH Lin CL Tsai WC Kung PT Chronic obstructive pulmonary disease and allied conditions is a strong independent risk factor for osteoporosis and pathologic fractures: a population-based cohort study. QJM (2015) 108(8):633–40. 10.1093/qjmed/hcv012 25614611

[20] Inoue D Watanabe R Okazaki R. COPD and osteoporosis: links, risks, and treatment challenges. Int J Chron Obstruct Pulmon Dis (2016) 11:637–48. 10.2147/COPD.S79638 27099481 PMC4820217

[21] Cavailles A Brinchault-Rabin G Dixmier A Goupil F Gut-Gobert C Marchand-Adam S Comorbidities of COPD. Eur Respir Rev (2013) 22(130):454–75. 10.1183/09059180.00008612 24293462 PMC9639181

[22] Tzouvelekis A Siafakas N Bouros D Comorbidities and chronic obstructive pulmonary disease: is there a place for lung fibrosis? Am J Respir Crit Care Med (2013) 188(11):1367 10.1164/rccm.201305-0927LE 24289776

[23] Chen W Thomas J Sadatsafavi M FitzGerald JM. Risk of cardiovascular comorbidity in patients with chronic obstructive pulmonary disease: a systematic review and meta-analysis. Lancet Respir Med (2015) 3(8):631–9. 10.1016/S2213-2600(15)00241-6 26208998

[24] Watz H Waschki B Boehme C Claussen M Meyer T Magnussen H. Extrapulmonary effects of chronic obstructive pulmonary disease on physical activity: a cross-sectional study. Am J Respir Crit Care Med (2008) 177(7):743–51. 10.1164/rccm.200707-1011OC 18048807

[25] Rana JS Mittleman MA Sheikh J Hu FB Manson JE Colditz GA Chronic obstructive pulmonary disease, asthma, and risk of type 2 diabetes in women. Diabetes Care (2004) 27(10):2478–84. 10.2337/diacare.27.10.2478 15451919

[26] Lange P Groth S Kastrup J Mortensen J Appleyard M Nyboe J Diabetes mellitus, plasma glucose and lung function in a cross-sectional population study. Eur Respir J (1989) 2(1):14–9. 2651148

[27] Davis WA Knuiman M Kendall P Grange V Davis TM Fremantle Diabetes S. Glycemic exposure is associated with reduced pulmonary function in type 2 diabetes: the Fremantle Diabetes Study. Diabetes Care (2004) 27(3):752–7. 10.2337/diacare.27.3.752 14988297

[28] Lee CT Mao IC Lin CH Lin SH Hsieh MC. Chronic obstructive pulmonary disease: a risk factor for type 2 diabetes: a nationwide population-based study. Eur J Clin Invest (2013) 43(11):1113–9. 10.1111/eci.12147 24028296

[29] Mirrakhimov AE. Chronic obstructive pulmonary disease and glucose metabolism: a bitter sweet symphony. Cardiovasc Diabetol (2012) 11:132. 10.1186/1475-2840-11-132 23101436 PMC3499352

[30] Caughey GE Preiss AK Vitry AI Gilbert AL Roughead EE. Comorbid diabetes and COPD: impact of corticosteroid use on diabetes complications. Diabetes Care (2013) 36(10):3009–14. 10.2337/dc12-2197 23735725 PMC3781532

[31] Lahousse L Loth DW Joos GF Hofman A Leufkens HG Brusselle GG Statins, systemic inflammation and risk of death in COPD: the Rotterdam study. Pulm Pharmacol Ther (2013) 26(2):212–7. 10.1016/j.pupt.2012.10.008 23142156

[32] Wang MT Lo YW Tsai CL Chang LC Malone DC Chu CL Statin use and risk of COPD exacerbation requiring hospitalization. Am J Med (2013) 126(7):598–606.e2. 10.1016/j.amjmed.2013.01.036 23684060

[33] Saaresranta T Polo O Hormones and breathing. Chest (2002) 122(6):2165–82. 10.1378/chest.122.6.2165 12475861

[34] Klein I Ojamaa K Thyroid (neuro)myopathy. Lancet (2000) 356(9230):614 10.1016/S0140-6736(00)02601-5 10968432

[35] Chopra IJ. Clinical review 86: euthyroid sick syndrome: is it a misnomer? J Clin Endocrinol Metab (1997) 82(2):329–34. 10.1210/jcem.82.2.3745 9024211

[36] Siafakas NM Salesiotou V Filaditaki V Tzanakis N Thalassinos N Bouros D. Respiratory muscle strength in hypothyroidism. Chest (1992) 102(1):189–94. 10.1378/chest.102.1.189 1623751

[37] Martinez FJ Bermudez-Gomez M Celli BR. Hypothyroidism. A reversible cause of diaphragmatic dysfunction. Chest (1989) 96(5):1059–63. 10.1378/chest.96.5.1059 2805837

[38] Ali NS Nanji K. A review on the role of vitamin D in asthma. Cureus (2017) 9(5):e1288. 10.7759/cureus.1288 28680776 PMC5491340

[39] Heck S Al-Shobash S Rapp D Le DD Omlor A Bekhit A High probability of comorbidities in bronchial asthma in Germany. NPJ Prim Care Respir Med (2017) 27(1):28. 10.1038/s41533-017-0026-x 28432297 PMC5435094

[40] Karampitsakos T Gourgoulianis KI. Asthma-COPD overlap syndrome (ACOS): single disease entity or not? Could exhaled nitric oxide be a useful biomarker for the differentiation of ACOS, asthma and COPD? Med Hypotheses (2016) 91:20–3. 10.1016/j.mehy.2016.04.008 27142135

[41] Schatz M Hsu JW Zeiger RS Chen W Dorenbaum A Chipps BE Phenotypes determined by cluster analysis in severe or difficult-to-treat asthma. J Allergy Clin Immunol (2014) 133(6):1549–56. 10.1016/j.jaci.2013.10.006 24315502

[42] Aranceta-Bartrina J Perez-Rodrigo C Alberdi-Aresti G Ramos-Carrera N Lazaro-Masedo S. Prevalence of general obesity and abdominal obesity in the Spanish adult population (aged 25–64 years) 2014–2015: the ENPE study. Rev Esp Cardiol (Engl Ed) (2016) 69(6):579–87. 10.1016/j.rec.2016.02.009 27133458

[43] Chinellato I Piazza M Sandri M Peroni D Piacentini G Boner AL. Vitamin D serum levels and markers of asthma control in Italian children. J Pediatr (2011) 158(3):437–41. 10.1016/j.jpeds.2010.08.043 20870246

[44] Devereux G Wilson A Avenell A McNeill G Fraser WD A case-control study of vitamin D status and asthma in adults. Allergy (2010) 65(5):666–7. 10.1111/j.1398-9995.2009.02220.x 19845573

[45] Forrest KY Stuhldreher WL. Prevalence and correlates of vitamin D deficiency in US adults. Nutr Res (2011) 31(1):48–54. 10.1016/j.nutres.2010.12.001 21310306

[46] Baffi CW Wood L Winnica D Strollo PJ Jr Gladwin MT Que LG Metabolic syndrome and the Lung. Chest (2016) 149(6):1525–34. 10.1016/j.chest.2015.12.034 26836925 PMC4944780

[47] Leone N Courbon D Thomas F Bean K Jego B Leynaert B Lung function impairment and metabolic syndrome: the critical role of abdominal obesity. Am J Respir Crit Care Med (2009) 179(6):509–16. 10.1164/rccm.200807-1195OC 19136371

[48] Brumpton BM Camargo CA Jr Romundstad PR Langhammer A Chen Y Mai XM. Metabolic syndrome and incidence of asthma in adults: the HUNT study. Eur Respir J (2013) 42(6):1495–502. 10.1183/09031936.00046013 23845717

[49] Cottrell L Neal WA Ice C Perez MK Piedimonte G. Metabolic abnormalities in children with asthma. Am J Respir Crit Care Med (2011) 183(4):441–8. 10.1164/rccm.201004-0603OC 20851922 PMC3056222

[50] Sodoyez-Goffaux FR Sodoyez JC De Vos CJ. Insulin receptors in the fetal rat lung. A transient characteristic of fetal cells? Pediatr Res (1981) 15(9):1303–7. 10.1203/00006450-198109000-00014 7290780

[51] Unger RH. Minireview: weapons of lean body mass destruction: the role of ectopic lipids in the metabolic syndrome. Endocrinology (2003) 144(12):5159–65. 10.1210/en.2003-0870 12960011

[52] Ronmark E Andersson C Nystrom L Forsberg B Jarvholm B Lundback B. Obesity increases the risk of incident asthma among adults. Eur Respir J (2005) 25(2):282–8. 10.1183/09031936.05.00054304 15684292

[53] Sideleva O Suratt BT Black KE Tharp WG Pratley RE Forgione P Obesity and asthma: an inflammatory disease of adipose tissue not the airway. Am J Respir Crit Care Med (2012) 186(7):598–605. 10.1164/rccm.201203-0573OC 22837379 PMC3480522

[54] Guerre-Millo M. Adipose tissue hormones. J Endocrinol Invest (2002) 25(10):855–61. 10.1007/BF03344048 12508947

[55] Sutter SA Stein EM. The skeletal effects of inhaled glucocorticoids. Curr Osteoporos Rep (2016) 14(3):106–13. 10.1007/s11914-016-0308-1 27091558 PMC4861637

[56] Barnig C Veaudor M Gautier C Margelidon-Cozzolino V Pigearias B Devouassoux G [How to consider triggers and comorbid conditions in severe asthma in adults]. Presse Med (2016) 45(11):1030–42. 10.1016/j.lpm.2016.07.020 27544706

[57] Dam TT Harrison S Fink HA Ramsdell J Barrett-Connor E Osteoporotic Fractures in Men Research Group Bone mineral density and fractures in older men with chronic obstructive pulmonary disease or asthma. Osteoporos Int (2010) 21(8):1341–9. 10.1007/s00198-009-1076-x 19816753 PMC2895883

[58] Richy F Bousquet J Ehrlich GE Meunier PJ Israel E Morii H Inhaled corticosteroids effects on bone in asthmatic and COPD patients: a quantitative systematic review. Osteoporos Int (2003) 14(3):179–90. 10.1007/s00198-003-1398-z 12730758

[59] Israel E Banerjee TR Fitzmaurice GM Kotlov TV LaHive K LeBoff MS. Effects of inhaled glucocorticoids on bone density in premenopausal women. N Engl J Med (2001) 345(13):941–7. 10.1056/NEJMoa002304 11575285

[60] Wong CA Walsh LJ Smith CJ Wisniewski AF Lewis SA Hubbard R Inhaled corticosteroid use and bone-mineral density in patients with asthma. Lancet (2000) 355(9213):1399–403. 10.1016/S0140-6736(00)02138-3 10791523

[61] Sosa M Saavedra P Valero C Guanabens N Nogues X del Pino-Montes J Inhaled steroids do not decrease bone mineral density but increase risk of fractures: data from the GIUMO Study Group. J Clin Densitom (2006) 9(2):154–8. 10.1016/j.jocd.2005.11.005 16785074

[62] Kuan YC How SH Azian AA Liam CK Ng TH Fauzi AR. Bone mineral density in asthmatic patients on inhaled corticosteroids in a developing country. Ann Thorac Med (2012) 7(2):69–73. 10.4103/1817-1737.94522 22558010 PMC3339206

[63] de Vries F Pouwels S Lammers JW Leufkens HG Bracke M Cooper C Use of inhaled and oral glucocorticoids, severity of inflammatory disease and risk of hip/femur fracture: a population-based case-control study. J Intern Med (2007) 261(2):170–7. 10.1111/j.1365-2796.2006.01754.x 17241182

[64] Iordanidou M Paraskakis E Giannakopoulou E Tavridou A Gentile G Borro M Vitamin D receptor ApaI a allele is associated with better childhood asthma control and improvement in ability for daily activities. OMICS (2014) 18(11):673–81. 10.1089/omi.2014.0023 25353337

[65] Baeke F Takiishi T Korf H Gysemans C Mathieu C. Vitamin D: modulator of the immune system. Curr Opin Pharmacol (2010) 10(4):482–96. 10.1016/j.coph.2010.04.001 20427238

[66] Korn S Hubner M Jung M Blettner M Buhl R. Severe and uncontrolled adult asthma is associated with vitamin D insufficiency and deficiency. Respir Res (2013) 14:25. 10.1186/1465-9921-14-25 23432854 PMC3648461

[67] Gupta A Dimeloe S Richards DF Chambers ES Black C Urry Z Defective IL-10 expression and in vitro steroid-induced IL-17A in paediatric severe therapy-resistant asthma. Thorax (2014) 69(6):508–15. 10.1136/thoraxjnl-2013-203421 24347461

[68] Urashima M Segawa T Okazaki M Kurihara M Wada Y Ida H. Randomized trial of vitamin D supplementation to prevent seasonal influenza A in schoolchildren. Am J Clin Nutr (2010) 91(5):1255–60. 10.3945/ajcn.2009.29094 20219962

[69] Majak P Olszowiec-Chlebna M Smejda K Stelmach I Vitamin D supplementation in children may prevent asthma exacerbation triggered by acute respiratory infection. J Allergy Clin Immunol (2011) 127(5):1294–6. 10.1016/j.jaci.2010.12.016 21315433

[70] Yadav M Mittal K. Effect of vitamin D supplementation on moderate to severe bronchial asthma. Indian J Pediatr (2014) 81(7):650–4. 10.1007/s12098-013-1268-4 24193954

[71] Martineau AR Cates CJ Urashima M Jensen M Griffiths AP Nurmatov U Vitamin D for the management of asthma. Cochrane Database Syst Rev (2016) 9:CD011511. 10.1002/14651858.CD011511.pub2 27595415 PMC6457769

[72] Antoniou KM Margaritopoulos GA Tomassetti S Bonella F Costabel U Poletti V. Interstitial lung disease. Eur Respir Rev (2014) 23(131):40–54. 10.1183/09059180.00009113 24591661 PMC9487254

[73] Wells AU Denton CP Interstitial lung disease in connective tissue disease – mechanisms and management. Nat Rev Rheumatol (2014) 10(12):728–39. 10.1038/nrrheum.2014.149 25266451

[74] Fischer A du Bois R. Interstitial lung disease in connective tissue disorders. Lancet (2012) 380(9842):689–98. 10.1016/S0140-6736(12)61079-4 22901890

[75] Karampitsakos T Woolard T Bouros D Tzouvelekis A. Toll-like receptors in the pathogenesis of pulmonary fibrosis. Eur J Pharmacol (2017) 808:35–43. 10.1016/j.ejphar.2016.06.045 27364757

[76] Tzouvelekis A Tzilas V Papiris S Aidinis V Bouros D Diagnostic and prognostic challenges in idiopathic pulmonary fibrosis: a patient’s “Q and A” approach. Pulm Pharmacol Ther (2017) 42:21–4. 10.1016/j.pupt.2016.12.002 27979760

[77] Kim D Cho SK Choi CB Choe JY Chung WT Hong SJ Impact of interstitial lung disease on mortality of patients with rheumatoid arthritis. Rheumatol Int (2017) 37:1735–45. 10.1007/s00296-017-3781-7 28748423

[78] Mikolasch TA Garthwaite HS Porter JC. Update in diagnosis and management of interstitial lung disease. Clin Med (Lond) (2016) 16(Suppl 6):s71–8. 10.7861/clinmedicine.16-6-s71 27956445 PMC6329571

[79] Oldham JM Collard HR. Comorbid conditions in idiopathic pulmonary fibrosis: recognition and management. Front Med (2017) 4:123. 10.3389/fmed.2017.00123 28824912 PMC5539138

[80] Tzouvelekis A Wang R Herazo-Maya J Castro JP DeIuliis G Woolard T Thyroid hormone as a novel therapeutic agent in lung fibrosis through restoration of AECs mitochondrial homeostasis. Am J Respir Crit Care Med (2016) 193:A4537–A.

[81] Gribbin J Hubbard R Smith C. Role of diabetes mellitus and gastro-oesophageal reflux in the aetiology of idiopathic pulmonary fibrosis. Respir Med (2009) 103(6):927–31. 10.1016/j.rmed.2008.11.001 19058956

[82] Enomoto T Usuki J Azuma A Nakagawa T Kudoh S. Diabetes mellitus may increase risk for idiopathic pulmonary fibrosis. Chest (2003) 123(6):2007–11. 10.1378/chest.123.6.2007 12796182

[83] Lamas DJ Kawut SM Bagiella E Philip N Arcasoy SM Lederer DJ. Delayed access and survival in idiopathic pulmonary fibrosis: a cohort study. Am J Respir Crit Care Med (2011) 184(7):842–7. 10.1164/rccm.201104-0668OC 21719755 PMC3208648

[84] Kaddah S Ahmed S Lifestyle associated diseases and risk of pulmonary hypertension in patients with idiopathic pulmonary fibrosis. Egypt J Chest Dis Tuberc (2016) 65(1):127–33. 10.1016/j.ejcdt.2015.06.006

[85] Sherbini N Feteih MN Wali SO Alamoudi OS Al-Faifi SM Khalid I. Idiopathic pulmonary fibrosis in Saudi Arabia: demographic, clinical, and survival data from two tertiary care hospitals. Ann Thorac Med (2014) 9(3):168–72. 10.4103/1817-1737.134073 24987477 PMC4073575

[86] Oldham JM Kumar D Lee C Patel SB Takahashi-Manns S Demchuk C Thyroid disease is prevalent and predicts survival in patients with idiopathic pulmonary fibrosis. Chest (2015) 148(3):692–700. 10.1378/chest.14-2714 25811599 PMC4556122

[87] Nowinski A Puscinska E Goljan A Peradzynska J Bednarek M Korzybski D The influence of comorbidities on mortality in sarcoidosis: a observational prospective cohort study. Clin Respir J (2017) 11(5):648–56. 10.1111/crj.12398 26470754

[88] Saidenberg-Kermanac’h N Valeyre D Boissier MC Vitamin D supplementation in patients treated for sarcoidosis: controversy or consensus? Joint Bone Spine (2017) 84(5):521–3. 10.1016/j.jbspin.2017.03.003 28323225

[89] Press DM Siperstein AE Berber E Shin JJ Metzger R Monteiro R The prevalence of undiagnosed and unrecognized primary hyperparathyroidism: a population-based analysis from the electronic medical record. Surgery (2013) 154(6):1232–7; discussion 7–8. 10.1016/j.surg.2013.06.051 24383100

[90] Tomos IP Tzouvelekis A Aidinis V Manali ED Bouros E Bouros D Extracellular matrix remodeling in idiopathic pulmonary fibrosis. It is the ‘bed’ that counts and not ‘the sleepers’. Expert Rev Respir Med (2017) 11(4):299–309. 10.1080/17476348.2017.1300533 28274188

[91] Karampitsakos T Tzilas V Tringidou R Steiropoulos P Aidinis V Papiris SA Lung cancer in patients with idiopathic pulmonary fibrosis. Pulm Pharmacol Ther (2017) 45:1–10. 10.1016/j.pupt.2017.03.016 28377145

[92] Tzouvelekis A Bonella F Spagnolo P. Update on therapeutic management of idiopathic pulmonary fibrosis. Ther Clin Risk Manag (2015) 11:359–70. 10.2147/TCRM.S69716 25767391 PMC4354471

[93] Oldham J Demchuk C Ma S-F Huang Y Strek ME Noth I Hypothyroidism in patients with idiopathic pulmonary fibrosis. Am J Respir Crit Care Med (2014) 189:A1497–A.

[94] Idiopathic Pulmonary Fibrosis Clinical Research Network Raghu G Anstrom KJ King TE Jr Lasky JA Martinez FJ. Prednisone, azathioprine, and N-acetylcysteine for pulmonary fibrosis. N Engl J Med (2012) 366(21):1968–77. 10.1056/NEJMoa1113354 22607134 PMC3422642

[95] Hyldgaard C Hilberg O Bendstrup E. How does comorbidity influence survival in idiopathic pulmonary fibrosis? Respir Med (2014) 108(4):647–53. 10.1016/j.rmed.2014.01.008 24529739

[96] Garcia-Sancho Figueroa MC Carrillo G Perez-Padilla R Fernandez-Plata MR Buendia-Roldan I Vargas MH Risk factors for idiopathic pulmonary fibrosis in a Mexican population. A case-control study. Respir Med (2010) 104(2):305–9. 10.1016/j.rmed.2009.08.013 19782552

[97] Garber JR Cobin RH Gharib H Hennessey JV Klein I Mechanick JI Clinical practice guidelines for hypothyroidism in adults: cosponsored by the American Association of Clinical Endocrinologists and the American Thyroid Association. Endocr Pract (2012) 18(6):988–1028. 10.4158/EP12280.GL 23246686

[98] Vanderpump MP Tunbridge WM French JM Appleton D Bates D Clark F The incidence of thyroid disorders in the community: a twenty-year follow-up of the Whickham Survey. Clin Endocrinol (Oxf) (1995) 43(1):55–68. 10.1111/j.1365-2265.1995.tb01894.x 7641412

[99] Tzouvelekis A Yu G Herazo-Maya J Wang R Werneck de Castro JPS DeIuliis G Thyroid hormone inhibits pulmonary fibrosis through enhancement of mitochondrial function in alveolar epithelial cells. Eur Respir J (2016) 48(Suppl):60.

[100] Yu G Tzouvelekis A Wang R Herazo-Maya JD Ibarra GH Srivastava A Thyroid hormone inhibits lung fibrosis in mice by improving epithelial mitochondrial function. Nat Med (2017). 10.1038/nm.4447 29200204 PMC5760280

[101] Furukawa S Fujita T Shimabukuro M Iwaki M Yamada Y Nakajima Y Increased oxidative stress in obesity and its impact on metabolic syndrome. J Clin Invest (2004) 114(12):1752–61. 10.1172/JCI21625 15599400 PMC535065

[102] Rice JB White A Lopez A Conway A Wagh A Nelson WW Economic burden of sarcoidosis in a commercially-insured population in the United States. J Med Econ (2017) 20:1048–55. 10.1080/13696998.2017.1351371 28678623

[103] Baughman RP Teirstein AS Judson MA Rossman MD Yeager H Jr Bresnitz EA Clinical characteristics of patients in a case control study of sarcoidosis. Am J Respir Crit Care Med (2001) 164(10 Pt 1):1885–9. 10.1164/ajrccm.164.10.2104046 11734441

[104] Statement on sarcoidosis. Joint Statement of the American Thoracic Society (ATS), the European Respiratory Society (ERS) and the World Association of Sarcoidosis and Other Granulomatous Disorders (WASOG) adopted by the ATS Board of Directors and by the ERS Executive Committee, February 1999. Am J Respir Crit Care Med (1999) 160(2):736–55. 10.1164/ajrccm.160.2.ats4-99 10430755

[105] Baughman RP Culver DA Judson MA. A concise review of pulmonary sarcoidosis. Am J Respir Crit Care Med (2011) 183(5):573–81. 10.1164/rccm.201006-0865CI 21037016 PMC3081278

[106] Martusewicz-Boros MM Boros PW Wiatr E Roszkowski-Sliz K. What comorbidities accompany sarcoidosis? A large cohort (n=1779) patients analysis. Sarcoidosis Vasc Diffuse Lung Dis (2015) 32(2):115–20. 26278690

[107] Antonelli A Fazzi P Fallahi P Ferrari SM Ferrannini E. Prevalence of hypothyroidism and Graves disease in sarcoidosis. Chest (2006) 130(2):526–32. 10.1378/chest.130.2.526 16899854

[108] Antonelli A Ferrari SM Corrado A Di Domenicantonio A Fallahi P. Autoimmune thyroid disorders. Autoimmun Rev (2015) 14(2):174–80. 10.1016/j.autrev.2014.10.016 25461470

[109] Baughman RP Janovcik J Ray M Sweiss N Lower EE Calcium and vitamin D metabolism in sarcoidosis. Sarcoidosis Vasc Diffuse Lung Dis (2013) 30(2):113–20. 24071882

[110] Baughman RP Papanikolaou I. Current concepts regarding calcium metabolism and bone health in sarcoidosis. Curr Opin Pulm Med (2017) 23(5):476–81. 10.1097/MCP.0000000000000400 28598871

[111] Adams JS Singer FR Gacad MA Sharma OP Hayes MJ Vouros P Isolation and structural identification of 1,25-dihydroxyvitamin D3 produced by cultured alveolar macrophages in sarcoidosis. J Clin Endocrinol Metab (1985) 60(5):960–6. 10.1210/jcem-60-5-960 2984238

[112] Bell NH Stern PH Pantzer E Sinha TK DeLuca HF. Evidence that increased circulating 1 alpha, 25-dihydroxyvitamin D is the probable cause for abnormal calcium metabolism in sarcoidosis. J Clin Invest (1979) 64(1):218–25. 10.1172/JCI109442 312811 PMC372108

[113] Kuchay MS Mishra SK Bansal B Farooqui KJ Sekhar L Mithal A. Glucocorticoid sparing effect of zoledronic acid in sarcoid hypercalcemia. Arch Osteoporos (2017) 12(1):68. 10.1007/s11657-017-0360-1 28726113

[114] Valeyre D Prasse A Nunes H Uzunhan Y Brillet PY Muller-Quernheim J. Sarcoidosis. Lancet (2014) 383(9923):1155–67. 10.1016/S0140-6736(13)60680-7 24090799

[115] Gibbs CJ Peacock M. Hypercalcaemia due to sarcoidosis corrects with bisphosphonate treatment. Postgrad Med J (1986) 62(732):937–8. 10.1136/pgmj.62.732.937 3774726 PMC2419051

[116] Xu EY Schaefer WH Xu Q. Metabolomics in pharmaceutical research and development: metabolites, mechanisms and pathways. Curr Opin Drug Discov Devel (2009) 12(1):40–52. 19152212

[117] Nobakht MGBF Aliannejad R Rezaei-Tavirani M Taheri S Oskouie AA. The metabolomics of airway diseases, including COPD, asthma and cystic fibrosis. Biomarkers (2015) 20(1):5–16. 10.3109/1354750X.2014.983167 25403491

[118] Saude EJ Skappak CD Regush S Cook K Ben-Zvi A Becker A Metabolomic profiling of asthma: diagnostic utility of urine nuclear magnetic resonance spectroscopy. J Allergy Clin Immunol (2011) 127(3):e1–6. 10.1016/j.jaci.2010.12.1077 21377043

[119] Adamko DJ Nair P Mayers I Tsuyuki RT Regush S Rowe BH Metabolomic profiling of asthma and chronic obstructive pulmonary disease: a pilot study differentiating diseases. J Allergy Clin Immunol (2015) 136(3):571–80.e3. 10.1016/j.jaci.2015.05.022 26152317

[120] Cap P Chladek J Pehal F Maly M Petru V Barnes PJ Gas chromatography/mass spectrometry analysis of exhaled leukotrienes in asthmatic patients. Thorax (2004) 59(6):465–70. 10.1136/thx.2003.011866 15170025 PMC1747035

[121] Ubhi BK Riley JH Shaw PA Lomas DA Tal-Singer R MacNee W Metabolic profiling detects biomarkers of protein degradation in COPD patients. Eur Respir J (2012) 40(2):345–55. 10.1183/09031936.00112411 22183483

[122] McClay JL Adkins DE Isern NG O’Connell TM Wooten JB Zedler BK (1)H nuclear magnetic resonance metabolomics analysis identifies novel urinary biomarkers for lung function. J Proteome Res (2010) 9(6):3083–90. 10.1021/pr1000048 20408573

[123] Paige M Burdick MD Kim S Xu J Lee JK Shim YM. Pilot analysis of the plasma metabolite profiles associated with emphysematous chronic obstructive pulmonary disease phenotype. Biochem Biophys Res Commun (2011) 413(4):588–93. 10.1016/j.bbrc.2011.09.006 21925153 PMC3199021

[124] Cloonan SM Glass K Laucho-Contreras ME Bhashyam AR Cervo M Pabon MA Mitochondrial iron chelation ameliorates cigarette smoke-induced bronchitis and emphysema in mice. Nat Med (2016) 22(2):163–74. 10.1038/nm.4021 26752519 PMC4742374

[125] Zhao YD Yin L Archer S Lu C Zhao G Yao Y Metabolic heterogeneity of idiopathic pulmonary fibrosis: a metabolomic study. BMJ Open Respir Res (2017) 4(1):e000183. 10.1136/bmjresp-2017-000183 28883924 PMC5531310

[126] Kang YP Lee SB Lee JM Kim HM Hong JY Lee WJ Metabolic profiling regarding pathogenesis of idiopathic pulmonary fibrosis. J Proteome Res (2016) 15(5):1717–24. 10.1021/acs.jproteome.6b00156 27052453

[127] Kottmann RM Kulkarni AA Smolnycki KA Lyda E Dahanayake T Salibi R Lactic acid is elevated in idiopathic pulmonary fibrosis and induces myofibroblast differentiation via pH-dependent activation of transforming growth factor-beta. Am J Respir Crit Care Med (2012) 186(8):740–51. 10.1164/rccm.201201-0084OC 22923663 PMC3480515

[128] Xie N Tan Z Banerjee S Cui H Ge J Liu RM Glycolytic reprogramming in myofibroblast differentiation and lung fibrosis. Am J Respir Crit Care Med (2015) 192(12):1462–74. 10.1164/rccm.201504-0780OC 26284610 PMC4731722

[129] Bueno M Lai YC Romero Y Brands J St Croix CM Kamga C PINK1 deficiency impairs mitochondrial homeostasis and promotes lung fibrosis. J Clin Invest (2015) 125(2):521–38. 10.1172/JCI74942 25562319 PMC4319413

[130] Kobayashi K Araya J Minagawa S Hara H Saito N Kadota T Involvement of PARK2-mediated mitophagy in idiopathic pulmonary fibrosis pathogenesis. J Immunol (2016) 197(2):504–16. 10.4049/jimmunol.1600265 27279371

[131] Larson-Casey JL Deshane JS Ryan AJ Thannickal VJ Carter AB. Macrophage Akt1 kinase-mediated mitophagy modulates apoptosis resistance and pulmonary fibrosis. Immunity (2016) 44(3):582–96. 10.1016/j.immuni.2016.01.001 26921108 PMC4794358

[132] Xie N Cui H Ge J Banerjee S Guo S Dubey S Metabolic characterization and RNA profiling reveal glycolytic dependence of pro-fibrotic phenotype of alveolar macrophages in lung fibrosis. Am J Physiol Lung Cell Mol Physiol (2017) 313(5):L834–44. 10.1152/ajplung.00235.2017 28798256 PMC5792180

[133] Ryu C Sun H Gulati M Herazo-Maya J Chen Y Osafo-Addo A Extracellular mitochondrial DNA is generated by fibroblasts and predicts death in idiopathic pulmonary fibrosis. Am J Respir Crit Care Med (2017) 196:1571–81. 10.1164/rccm.201612-2480OC 28783377 PMC5754440

